# Non-vertebral *Veillonella* species septicemia and osteomyelitis in a patient with diabetes: a case report and review of the literature

**DOI:** 10.1186/1752-1947-8-365

**Published:** 2014-11-12

**Authors:** Fawzia Eida Al-Otaibi, Maha Mohammed Al-Mohizea

**Affiliations:** 1 King Khalid University Hospital, King Saud University, PO: 2925, Riyadh 11461, Saudi Arabia

**Keywords:** Diabetic foot, Osteomyelitis, *Veillonella* bacteremia

## Abstract

**Introduction:**

*Veillonella* is a nonfermentative, strictly anaerobic, Gram-negative coccus that forms part of the human gastrointestinal tract, mouth and vaginal flora. Like other anaerobic infection, *Veillonella* species usually are involved in polymicrobial processes, which make it difficult to determine their pathogenic role. Isolation of a clinically significant *Veillonella* species is rare and *V. parvula* is the most common one reported to cause infection in humans. The most frequently reported infection caused by *V. parvula* is osteomyelitis, almost always in association with bacteremia.

**Case presentation:**

Here, we describe a rare case of nonvertebral osteomyelitis and septicemia caused by *Veillonella* species in a 49-year-old Saudi man with diabetes. Initial treatment with ciprofloxacin was associated with treatment failure and poor response. Identification of the organism was essential for the selection of appropriate treatment. There have been only seven previous reports of *Veillonella* vertebral osteomyelitis and one report of *V. parvula* foot osteomyelitis with sepsis in the literature. This is the second case of *Veillonella* nonvertebral osteomyelitis associated with septicemia reported to date.

**Conclusions:**

*Veillonella* species should be considered a true pathogen in diabetic patients with osteomyelitis and those with underlying immune suppression, particularly if the organism is isolated from blood. The isolation of those obligate anaerobes from blood may signal the presence of severe underlying disease and the probable need for timely surgical intervention.

## Introduction

*Veillonella* is a nonfermentative, small, nonmotile, strictly anaerobic, Gram-negative cocci that form part of the normal flora of the oral, respiratory, intestinal, and female genital tracts [1]. They are usually recovered as part of a polymicrobial infection and they are often regarded as a contaminant. However, they have been isolated in pure culture from various sterile body sites such as sinuses, lungs, liver, central nervous system, heart, and bone. *Veillonella parvula* is the most common clinically significant species and is infrequently grow from blood cultures. We report here a case of *Veillonella* species bacteremia associated with osteomyelitis in which pure *Veillonella* species was isolated from blood culture. This is the second reported case of *Veillonella* nonvertebral osteomyelitis, which illustrates the pathogenic potential of this organism in causing invasive infection in diabetic patients.

## Case presentation

**Figure 1:**
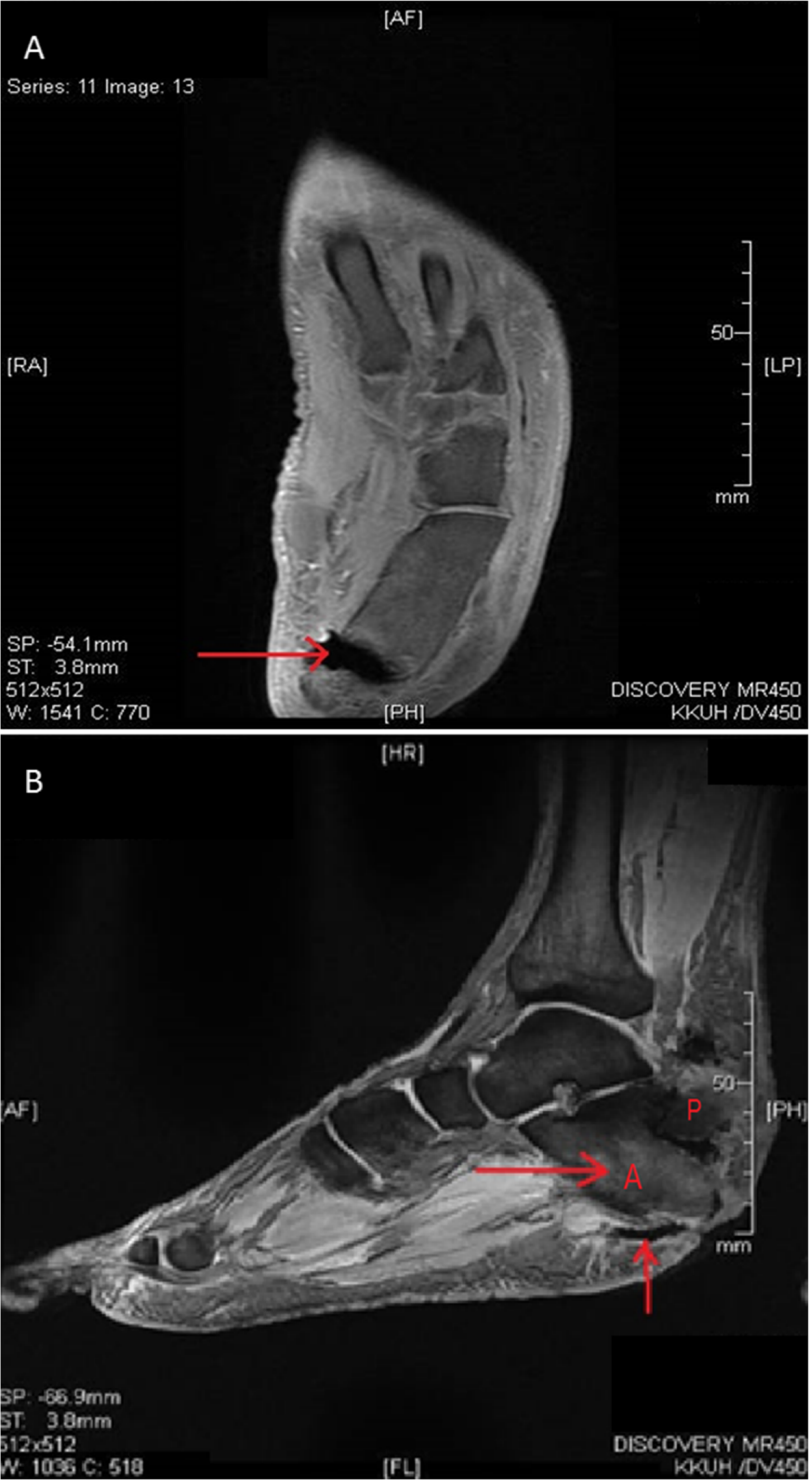
**Left foot magnetic resonance imaging. (A)** A left foot MRI scan revealed a fracture in the calcaneus. **(B)** The calcaneus bone is separated into two segments ((A) and (P)) with evidence of anterior segment osteomyelitis and a deep, infected ulcer (arrows).

**Figure 2:**
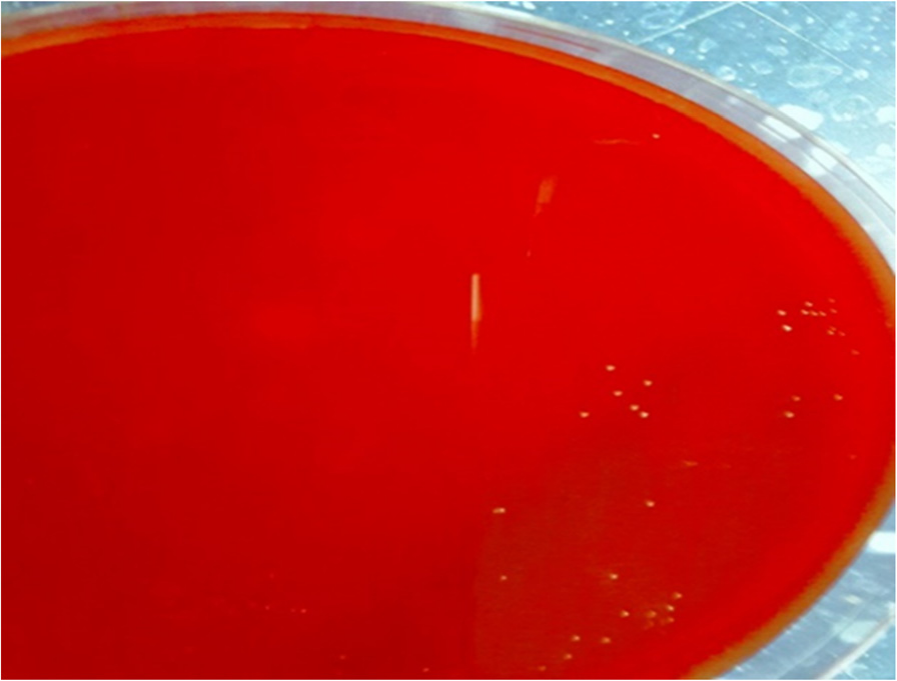
**Blood agar plate after 48 hours of anaerobic incubation showing tiny colonies of *Veillonella *** **organism.**

**Figure 3:**
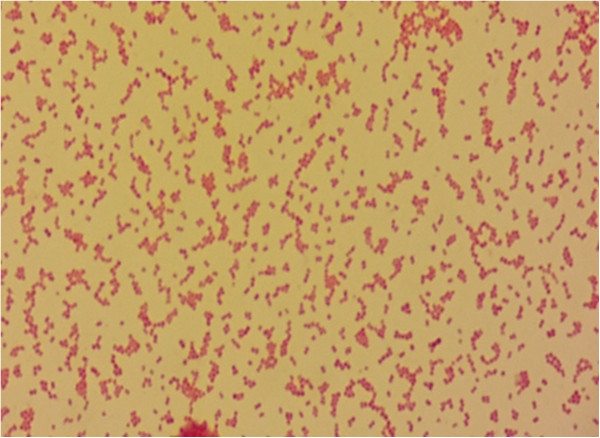
Gram stain from anaerobic culture plate showing small Gram-negative cocci.

A 49-year-old Saudi man, a known case of insulin-dependent diabetes mellitus (IDDM), hypertension and ischemic heart disease, presented to the emergency department with a three-day history of fever, chills and ankle pain after trauma to his left foot. He reported a nonhealing ulcer on his left heel following a hot surface injury 20 days prior to his presentation. On examination, our patient looked ill and septic, with minimal ambulation. His temperature was 38.9°C and his blood pressure and pulse were 140/90mmHg and 108 beats/minutes respectively. A lower limb examination revealed bilateral decrease of sensation and a foot ulcer on his left heel. Careful examination of his left foot showed a 7×5cm deep ulcer on the lateral side that looked badly infected with change of skin color and profuse pus discharge. Our patient was seen by the orthopedic surgeon and an urgent extensive wound debridement involving the bone was undertaken. Blood, pus and bone tissue specimens were collected and sent to the microbiology laboratory for culture and sensitivity testing. A laboratory examination showed a white cell count (WBC) of 29×10^9^g/L with 90% neutrophils, hemoglobin of 6.9g/L, and an erythrocyte sedimentation rate (ESR) of 97mm/h. His serum creatinine was 123umol/L, and urea mmol/L 8.9. His liver function test results were within normal limits except for serum alkaline phosphatase (ALP), which was raised (785IU/L). A left foot X-ray revealed soft tissue swelling, gas formation and fracture of the calcaneus bone. A left foot magnetic resonance imaging (MRI) scan revealed a calcaneus fracture with high suspicion of calcaneus anterior fragment osteomyelitis (Figure 1). An MRI scan of his right foot revealed mild neuropathic arthropathy (Charcot joint) with no evidence of osteomyelitis. Our patient was started empirically on intravenous ciprofloxacin 400mg twice daily, and intravenous clindamycin 600mg eight hourly. During the first three days after starting treatment, our patient showed no clinical improvement and he continued to run a low-grade fever. A pus culture grew *Escherichia coli* sensitive to ciprofloxacin, imipenem, meropenem, gentamicin and *Enterococcus avium* sensitive to ampicillin. A bone and tissue biopsy grew *Morganella morganii* sensitive to gentamicin, imipenem and resistant to ciprofloxacin, ampicillin, amoxicillin-clavulanic acid, piperacillin/tazobactam and cotrimoxazole. No growth occurred on the anaerobic plates despite prolonged incubation. Based on culture results, the initial antibiotics were suspended and our patient was started on imipenem 500mg intravenously (IV) six hourly, vancomycin 1g IV 12 hourly, and colistin 2 million units IV eight hourly. The blood culture obtained before treatment initiation, which was inoculated in an anaerobic bottle (Bactec Lytic/10, Anaerobic/F, Beckton Dickinson, Franklin Lakes, NJ, USA) and processed by an automated blood culture system (Bactec FX, Beckton Dickinson), grew very scanty Gram-negative cocci within 18 hours shown on Gram stain. The blood then was subcultured on Trypticase soy agar with sheep blood (BBL Microbiology Systems, Cockeysville, MD, USA) and incubated at 35°C in 5% CO2, in an anaerobic GasPak™ jar (BBL Microbiology Systems). After 48 hours of anaerobic incubation, the blood agar grew slow-growing tiny colonies (Figure 2) that showed Gram-negative cocci on Gram stain (Figure 3). The organism was identified by the Vitek 2 automated system (bioMérieux, Marcy-l'Étoile, France), as *Veillonella* species with a 99% probability rate. It was sensitive to imipenem (1.5μg/ml), clindamycin (0.047μg/ml), ceftriaxone (8μg/ml) and colistin, and resistant to penicillin (>32μg/ml), vancomycin, erythromycin and metronidazole. Further identification to species level and genotyping was unable to be performed, as the subcultured isolates failed to grow and the significant time delay before attempting to reisolate the organisms from the blood culture bottle rendered them nonviable. Over the subsequent five days, our patient’s condition worsened with a high temperature (38.9°C), rigors, a high WBC and ESR count. The wound was reevaluated by the surgical team and a decision of above-knee amputation was taken. Colistin and vancomycin were discontinued and imipenem was continued for four weeks. On follow-up, our patient showed gradual improvement of his condition and was discharged.

## Discussion

*Veillonella* species are rare causes of serious infections such as meningitis [2], endocarditis [3], obstructive pneumonitis [4], prosthetic joint infection [5], and bacteremia [6]. Bacteremia has been reported almost always in association with an underlying infection such as osteomyelitis. There have been seven previous reports of vertebral osteomyelitis [7-13] and one report of foot cellulitis and osteomyelitis in a middle-aged woman associated with sepsis caused by *V. parvula*[14] (Table 1). Our report is considered to be the second report of V*eillonella* nonvertebral osteomyelitis associated with bacteremia. Borchardt [14] reported the first case of nonvertebral osteomyelitis in which a 54-year-old Indian diabetic woman developed foot cellulitis and toe osteomyelitis after attempting to shave a callus on her right foot two months before presentation. The patient recovered completely after excision of the phalanx and metatarsal bones along with four weeks of intravenous penicillin treatment. *V parvula* was isolated from the excised tissue and the blood culture. In five of the previously reported clinical infections, the patients were previously healthy and the portal of entry was not identified. Isner-Horobeti [11] reported on a 27-year-old man with unknown risk factors for infection, who presented with an L4 to L5 spondylodiscitis. The patient was cured after a prolonged course (11 weeks) of intravenous followed by oral penicillin. In another report by Bongaerts [9]*V. parvula* was isolated from the spine (T12 to L1) of a previously healthy 74-year-old man who was treated with six weeks of intravenous penicillin. Similar cases were reported by Hidalgo [10] and Kishen *et al.*[12] of *Veillonella* spondylodiscitis in older patients without any risk factors. The outcome of the patients was favorable after antibiotic treatment alone or combined with surgical management. On the other hand, colonoscopy was considered by Marriott [7] as a possible source of entry of *V. parvula* causing bacteremia and lumbar discitis in a 55-year-old man who had undergone small intestinal and rectal biopsies eight weeks prior to presentation. In another report by Barnhart [13], postoperative *Veillonella* osteomyelitis of the cervical spine was reported from a 31-year-old man who had suffered a cervical fracture at the level of the fourth and fifth cervical vertebrae two months before presentation. The possible source of entry of the organism was thought to be the esophageal perforation that had occurred during surgery. *Veillonella* was isolated repeatedly from a retropharyngeal soft tissue abscess, which was drained followed by parenteral penicillin administration for six weeks. The best method for identification of *Veillonella* strains at the species level requires direct sequencing of the 16S rRNA gene [15]. Conventional phenotypic and biochemical testing does not provide adequate discrimination between species. The pathogenic role of these anaerobes has not been established. However, previously published reports demonstrated their role as a true pathogen associated with fatal overwhelming septicemia [6]. In our case, in which *Veillonella* species was isolated in pure culture from blood in a patient with signs of severe diabetic wound infection illustrates the pathogenic role of this microorganism. The strictly anaerobic organism failed to grow from the wound probably due to improper sample collection and transport supportive of anaerobic culture. In addition, the presence of other rapidly growing aerobic organisms as part of the polymicrobial infection might interfere with their recovery from wound infection. *Veillonella* is usually vancomycin, tetracycline, aminoglycosides, and ciprofloxacin resistant and infection typically responds well to therapy with penicillin. Other antimicrobial agents to which the organism is usually susceptible *in vitro* include cephalosporins, clindamycin, metronidazole, and chloramphenicol. There are no clear treatment recommendations in the literature due to the scarce number of reports on *Veillonella* as a pathogen associated with invasive infection. However, the previous few reports showed good response to antibiotics such as penicillin, cephalosporins, chloramphenicol, clindamycin and metronidazole [16-20]. Metronidazole was suggested by Warner *et al.*[16] as an effective drug for the management of serious infections like bacteremia, brain abscess, and meningitis. The isolate from our case showed unusual high minimum inhibitory concentration (MIC) to penicillin (>32μg/ml). However, in a recent study *Veillonella* species isolated from the oral cavities of humans demonstrated a high level of resistance to penicillin G (MIC, 2μg/ml) [21] and reduced susceptibility to ampicillin or amoxicillin. Reports on the management of infections caused by *Veillonella* isolates demonstrating high MIC to penicillin is lacking due to limited published studies on the susceptibility of *Veillonella* species to different antimicrobial agents, particularly penicillin, and their clinical effectiveness.

**Table 1:** **Previously reported cases of osteomyelitis caused by *Veillonella***

**Number of patients (reference)**	**Age (year)/sex**	**Culture result**		**Underlying disease/risk factors**	**Type of bone/soft tissue**	**Antibiotic therapy**
		**Blood**	**Bone/disc**			
**1. (This study)**	49/M	*Veillonella sp*.	NR	Diabetes mellitus and diabetic foot	Foot bone (Calcenous)	Imipenem
**2. Marriott **[7]	55/M	*V. parvula*	*V. parvula*	Colonoscopy	Lumbar spine discitis	Ceftriaxone
**3. Singh **[8]	61/F	*V. parvula*	*V. parvula*	Sjogren’s syndrome, xerostomia-	Thora columbar spine	Ceftriaxone
**4. Bongaerts **[9]	74/M	NR	*V. parvula*	None	Spine	Penicillin
**5. Hidalgo **[10]	70/M	NR	*V. parvula*	None	Spine	US
**6** ***. *** **Isner-Horobeti **[11]	27/M	NR	*Veillonella sp.*	None	Lumbar spondylodiscitis	Amoxicillin
**7. Kishen **[12]	76/F	*Veillonella sp.*	*Veillonella sp.*	None	Lumbar spondylodiscitis and paraspinal space abscesses	Spinal surgery + Cefotaxime/metronidazole
**8. Barnhart **[13]	31/M	NR	*Veillonella sp.*	Cervical vertebral fracture/Post cervical spine fusion	Cervical spine and retropharyngeal abscess	Penicillin G + abscess drainage
**9. Borchardt **[14]	54/F	*V. parvula*	*V. parvula*	Diabetes mellitus and foot shaving injury	Phalanx and metatarsal bone and cellulitis	Excision of infected bone + penicillin

NR, not reported; US, unspecified; M, male; F, female.

## Conclusion

*Veillonella* species should be considered a true pathogen in diabetic patients with osteomyelitis and those with underlying immune suppression, particularly if the organism is isolated from blood. The isolation of those obligate anaerobes from blood may signal the presence of severe underlying disease and the probable need for timely surgical intervention.

## Consent

Written informed consent was obtained from the patient for publication of this case report and any accompanying images. A copy of the written consent is available for review by the Editor-in-Chief of this journal.

## Abbreviations

ALP: alkaline phosphatase; ESR: erythrocyte sedimentation rate; IDDM: insulin-dependent diabetes mellitus; IV: intravenous; MIC: minimum inhibitory concentration; MRI: magnetic resonance imaging; NR: not reported; US: unspecified; USA: United States of America; WBC: white cell count.

## Competing interests

The authors declare that they have no competing interests.

## Authors’ contributions

FEA and MMA were involved in collecting the data and writing and critically reviewing the manuscript. Both authors read and approved the final manuscript.
